# Association of *in vitro* fertilization with maternal and perinatal outcomes among pregnant women with active tuberculosis: A retrospective hospital-based cohort study

**DOI:** 10.3389/fpubh.2022.1021998

**Published:** 2022-10-17

**Authors:** Lu Xia, Peierdun Mijiti, Xu-Hui Liu, Zhi-Dong Hu, Xiao-Yong Fan, Shui-Hua Lu

**Affiliations:** ^1^Shanghai Public Health Clinical Center, Fudan University, Shanghai, China; ^2^The Key Laboratory of Medical Molecular Virology of Ministries of Education and Health, School of Basic Medical Science, Fudan University, Shanghai, China; ^3^Department of Pulmonary Medicine, The Third People's Hospital of Shenzhen, Shenzhen, China; ^4^National Clinical Research Center for Infectious Diseases, Shenzhen, China

**Keywords:** tuberculosis, *in vitro* fertilization, immunity, miliary TB, perinatal outcome

## Abstract

**Background:**

Study on effect of fertilization methods on maternal and perinatal outcomes with respect to TB during pregnancy was scarce. This study aimed to analyze maternal and perinatal outcomes in active TB cases after *in vitro* fertilization (IVF) treatment vs. normal pregnancy.

**Methods:**

Clinical data of 80 pregnant women with active TB hospitalized at Shanghai Public Health Clinical Center between June 1st, 2014 and November 30th, 2020 were extracted and retrospectively analyzed. History of receiving IVF was recorded at admission and its association with maternal and perinatal outcomes were assessed using multivariable logistic regression models with adjustment for potential confounders.

**Results:**

Of the 80 pregnant women with active TB, 28 (35.0%) received IVF treatment and 52 (65.0%) did not receive IVF treatment. After adjusting for potential confounders, receiving IVF was associated with worse maternal and perinatal outcomes, including maternal criticality (21.4 vs. 2.0%, adjusted OR = 28.3, *P* = 0.015), miliary TB (89.3 vs. 13.5%, adjusted OR = 75.4, *P* < 0.001), TB meningitis (32.1 vs. 7.7%, adjusted OR = 6.2, *P* = 0.010), and perinatal mortality (64.3 vs. 28.8%, adjusted OR = 9.8, *P* = 0.001).

**Conclusion:**

The additional risk of TB to women receiving IVF treatment is a public health challenge specific to countries with a high tuberculosis burden. Increased awareness of latent tuberculosis infection in women receiving IVF treatment is needed.

## Introduction

Tuberculosis (TB) is a communicable disease caused by *Mycobacterium tuberculosis* (MTB). Approximately 2 billion people were infected by MTB globally in 2020 and 5–10% will develop tuberculosis (TB) in their lifetime (1). It is estimated that about 217 thousand active TB cases among pregnancy women in 2011 (2). In high TB-burden regions, TB is considered as a leading non-obstetric cause of mortality in women of reproductive age (3, 4), and is also associated with adverse perinatal outcomes, including preterm birth, low birth weight, birth asphyxia and perinatal death. Studies has shown that pregnancy increases the susceptibility to new infections and reactivation of TB (3, 5), partly owing to the effect of endogenous progesterone (6, 7). Progestogens may suppress host immunity with a dose-dependent effect on the Th1/Th2 response and thus reduce T-cell proliferation (6). The host immune response, especially the equilibrium between T-helper 1 (Th1) and T-helper 2 (Th2) cells, is critical for determining the outcome of MTB infection or disease (8–10).

*In vitro* fertilization (IVF) is the most widely used assisted reproductive technology, and progestogen is administered during the IVF procedure (11). The progestogen dose used during IVF is approximately 2–4-fold or higher than that of the normal supplement administered to reduce the risk of preterm birth or increase maternal-fetal tolerance (12, 13). The development of TB after IVF treatment reportedly results in severe maternal complications (such as progressive respiratory failure, miliary TB, or tuberculous meningitis) and unfavorable perinatal outcomes (14–17). Therefore, it is reasonable to assume that exogenous progestogen used in IVF may induce excessive immunosuppressive effects and result in adverse consequences when overlapped with tuberculosis.

To date, the association between IVF treatment and the outcome of TB-in-pregnancy is unclear. No published study has compared complications or outcomes of TB patients receiving IVF with those not receiving IVF. To assess the association between IVF treatment and maternal and perinatal outcomes in active TB cases during pregnancy, we conducted a retrospective study based on registered data on electronic medical record (EMR) system.

## Methods

### Study design and participants

This retrospective cohort study was conducted at Shanghai Public Health Clinical Center (SHPHCC), the only inpatient center for pregnant women with tuberculosis in Shanghai. In the past decade, almost all patients with active TB during pregnancy or postpartum that require hospitalization service in Shanghai were registered at this center.

We screened all in-patients with TB-in-pregnancy record at SHPHCC from June 1, 2014 to November 30, 2020. Those who met inclusion and exclusion criteria were considered eligible. Inclusion criteria included: (1) 18–42 years old; (2) developed active TB during pregnancy or within 6 weeks after delivery. Exclusion criteria include the following: (1) those who have developed TB before pregnancy according to clinical assessment; (2) those who failed to receive routine anti-TB treatment for any reason, such as severe adverse effect of drugs, or rejected to receive anti-TB therapy; (3) those without complete medical records.

An active TB case was defined as both bacteriologically (a valid specimen with positive results on smear microscopy, culture, histopathology, or a Food and Drug Administration-approved nucleic acid amplification test, whichever was available) and clinically (diagnosed as TB with a decision to treat by experienced clinicians without a bacteriologically confirmation) positive (18).

### Data collection

Demographic and clinical data of all eligible study participants were extracted from the electronic medical record (EMR) system of SHPHCC. These data included age, symptoms on admission (such as cough, fever, night sweats, weight loss, vaginal bleeding, pleural effusion, and progressive respiratory failure), TB diagnostic test results (sputum smear, sputum culture, interferon-gamma release assay, nucleic acid amplification tests), chest radiography findings, drug susceptibility tests results, anti-TB treatment regimen, whether HIV positive and CD4+ cell counts and maternal/perinatal outcomes. Data on dates of first TB-related symptoms, TB diagnosis, and pregnancy were extracted as well. The history of IVF treatment was determined by checking medical records or via a telephonic interview. If any clinical information was missing, we conducted a telephonic interview to collect the required data.

### Definition of variables

Maternal outcomes include obstetric complications (whether to develop preeclampsia or criticality at the time of delivery to 6 weeks postpartum) and TB outcomes (whether to develop TB-related symptoms, systemic dissemination, or death). Maternal criticality is defined as a life-threatening condition, including acute respiratory distress syndrome, severe eclampsia, hemorrhage, or loss of consciousness, requiring comprehensive care and constant monitoring.

Perinatal outcomes include preterm birth rate, perinatal mortality, and TB outcome (whether to develop TB after birth).

Delay in diagnosis is quantified using the days from the first TB symptom to the diagnosis of TB. The time to the first TB symptom was calculated using gestational weeks from conception to the first TB symptom. Patients with a medical record of IVF treatment or confirmed history via the telephonic interview were categorized into the treatment group (IVF-TB) and those without a record or confirmation into the control group (not IVF-TB).

### Ethics statement

This study procedure was reviewed and approved by the Ethics committee of SHPHCC (2020-S203-01) and a waiver of informed consent was obtained.

### Statistical analysis

Statistical analyses were performed using IBM SPSS Statistics version 22. Categorical variables are presented as percentage (%), and continuous variables are presented as mean ± standard deviation or median (interquartile range). Differences in categorial variables were analyzed using the chi-square test or Fisher's exact test, whereas differences in continuous variables were analyzed using Student's *t*-test or the Mann–Whitney U test. Logistic regression analysis was performed to determine the association between patient characteristics and each maternal complications, and between IVF treatment and each perinatal outcome. Crude and adjusted odds ratios and their 95% confidence intervals (CIs) were estimated. *P* < 0.05 indicated statistical significance in both univariate and multivariate analysis.

## Results

### Characteristics of participants

**Figure 1:**
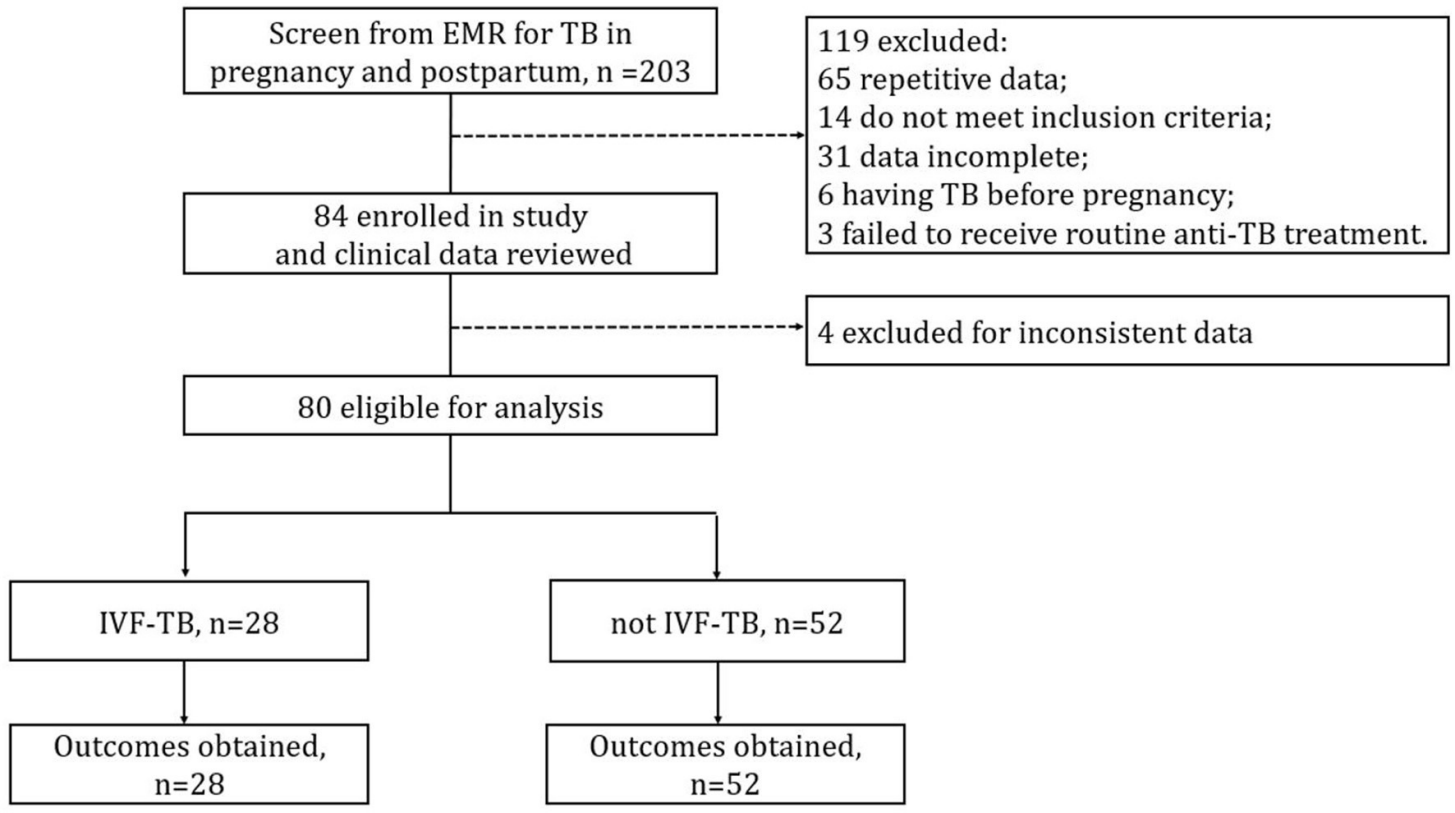
Flow chart of participants and data collection. EMR, electronic medical record; IVF, *in vitro* fertilization.

A total of 98 patients with TB during pregnancy or postpartum were hospitalized at SHPHCC during the study period. Of them, 80 had complete data and were included in the final analysis; 18 were excluded either owing to ineligibility or incomplete medical records (Figure 1). The 80 participants a mean age of 28.05 ± 4.81 years; 59 (73.8%) had bacteriologically confirmed TB, 7 (8.8%) reported a history of TB, 77 (77/79, 97.5%) tested positive on interferon-gamma release assays (IGRAs), and none tested positive for HIV. All participants received anti-TB treatment under experienced clinicians' guidance, and none were diagnosed with MDR-TB (resistant to both isoniazid and rifampicin). 28 (35.0%) patients reported a history of IVF treatment, and 67.9% (19/28) of them had experienced fallopian tube obstruction before IVF treatment.

The median time to the first TB symptom in IVF treatment group was less than that in control group (13 vs. 22 gestational weeks, *P* < 0.001). The CD4+ T cell count was lower in treatment group than in control group (337 ± 152 vs. 454 ± 237, *P* = 0.086). There was no statistical difference in age, delay in diagnosis, TB history, and percentage of bacteriological confirmation between the two groups (Table 1).

**Table 1:** Characteristics of study participants.

	**IVF-TB**	**Non-IVF-TB**	***P-value***
Cases (n)	28	52	
Age (years, mean ± SD)	29.46 ± 4.03	27.29 ± 5.05	0.053
**Reasons for IVF (%)**			
Fallopian tube obstruction	19 (67.9)	-	-
Male infertility	1 (3.6)	-	-
Unknown	8 (28.6)	-	-
Time to first TB symptom(s) onset (gestational weeks, median ± IQR)	13 (8-17.75)	22 (15-31.75)	<0.001
Delay in TB diagnosis (days, median ± IQR)	14.5 (7.25-30)	14 (7-28.5)	0.466
Onset postpartum TB (%)	1 (3.6)	5 (9.6)	0.659
History of TB (%)	4 (14.3)	3 (5.8)	0.232
**Diagnosis of TB**			
Bacteriologically confirmed TB (%)	22 (78.6)	37 (71.2)	0.597
Clinically diagnosed TB (%)	6 (21.4)	15 (28.8)	
Smear positive (%)	16 (57.1)	23 (44.2)	0.350
Culture positive (%)	18 (64.3)	32/51 (62.7)	1.000
IGRA positive (%)	28/28 (100)	49/51 (96.1)	0.537
CD4+ T cell count (cell/μL, mean ± SD)	337 ± 152 (*n* = 15)	454 ± 237 (*n* = 32)	0.086

IGRA, interferon-gamma release assay; IQR, interquartile range; TB, tuberculosis.

### Maternal outcomes in association with receiving IVF treatment

Obstetric complications, such as vaginal bleeding (46.4 vs. 1.9%, *P* < 0.001) and maternal criticality (21.4 vs. 2.0%, *P* = 0.007) were more common among participants receiving IVF treatment compared to those without receiving IVF. TB-related symptoms or complications were also more common in treatment group, miliary TB (89.3 vs. 13.5%, *P* < 0.001), TB meningitis (32.1 vs. 7.7%, *P* = 0.008), fever > 38.2°C (92.9 vs. 51.9%, *P* < 0.001), except pleural effusion (3.6 vs. 36.5%, *P* = 0.001). None TB-related maternal death was reported in this study.

After adjustment of potential confounders (age, delay in diagnosis, and MTB culture result), receiving IVF treatment was associated with a higher risk of vaginal bleeding (adjusted OR = 47.6), maternal criticality (adjusted OR = 28.3), fever > 38.2°C (adjusted OR = 16.7), cough (adjusted OR = 2.3), miliary TB (adjusted OR = 75.4), and TB meningitis (adjusted OR = 6.2). (Table 2).

**Table 2:** Association between IVF status and maternal outcome among TB-in-pregnancy women.

	**IVF-TB**	**Non-IVF-TB**	OR^*^	**95% CI**	***P*** **-value (crude)**	adjusted OR^**^	**95% CI**	***P*** **-value**
	**(*n* = 28)**	**(*n* = 52)**						**(adjusted)**
**Obstetric complications (%)**								
Vaginal bleeding	13 (46.4)	1 (1.9)	44.2	5.3–366.0	< 0.001	47.6	5.2–439.6	0.001
Maternal criticality	6 (21.4)	1 (2.0)	13.9	1.6–122.4	0.007	28.3	1.9–417.2	0.015
Preeclampsia	0 (0)	0 (0)	-		-	-		-
**TB outcomes (%)**								
Fever > 38.2°C	26 (92.9)	27 (51.9)	12	2.6–56.0	< 0.001	16.7	3.2–87.7	0.001
Cough	21 (75.0)	30 (57.7)	2.2	0.8–6.1	0.149	2.3	0.8–6.7	0.135
Pleural effusion	1 (3.6)	19(36.5)	0.06	0.01–0.51	0.001	0.05	0.01–0.42	0.006
Miliary TB	25 (89.3)	7 (13.5)	53.6	12.7–225.7	< 0.001	75.4	13.7–415.2	<0.001
TB meningitis	9 (32.1)	4 (7.7)	6	1.6–21.9	0.008	6.2	1.5–24.8	0.01
Death	0 (0)	0 (0)	-		-	-		-

* Odds ratio for IVF treatment vs. no IVF treatment by univariate analysis;

** Multivariate regression was applied after adjusting for age, delay in diagnosis, and culture result.

### Perinatal outcomes in association with receiving IVF treatment

Perinatal mortality was significantly higher in the treatment group (64.3 vs. 28.8%, *P* = 0.008). After adjusting for age, delay in diagnosis, and sputum MTB culture, IVF was significantly associated with increased infant mortality (adjusted OR = 5.8, *P* = 0.002). Fourteen participants chose to receive induced abortion. After induced abortion was excluded in the analysis, IVF was still associated with increased infant mortality (adjusted OR = 9.8, *P* = 0.001) (Table 3).

**Table 3:** Association between IVF status and perinatal outcome among TB-in-pregnancy women.

	**IVF-TB**	**Non-IVF-TB**	OR^*^	**95% CI**	***P*** **-value (crude)**	Adjusted OR^**^	**95% CI**	***P*** **-value (adjusted)**
	**(*n* = 28)**	**(*n* = 52)**						
Mortality (%)	18 (64.3)	15 (28.8)	4.4	1.7–11.8	0.004	9.8	2.6–36.8	0.001
Spontaneous abortion	13 (46.4)	6 (11.5)	-		-	-		-
Induced abortion	5 (17.9)	9 (17.3)	-		-	-		-
Preterm birth (alive) (%)	2 (7.1)	3 (5.8)	-		-	-		-
Normal birth (%)	8 (28.6)	34 (65.4)	-		-	-		-
Developed TB after birth^***^ (%)	0 (0)	0 (0)	-		-	-		-

* Odds ratio for IVF treatment vs. no IVF treatment by univariate analysis;

** Multivariate regression was applied after adjusting for age, delay in diagnosis, culture result, and induced abortion according to the patient's will.

*** These data came from medical records or telephone review conducted at least 1 year after delivery.

## Discussion

IVF treatment is widely used to help with fertility and assist with the conception of a child. The risks of adverse perinatal outcomes associated with IVF treatment are generally mild, with a favorable success rate (19–21). However, TB infection, especially active TB may complicate this. Based on a retrospective study, we have proved that receiving IVF treatment is a potential risk factor associated with severe maternal complications and worse perinatal outcomes in TB-in-pregnancy patients. In areas with high tuberculosis prevalence, such as China and India, the number of pregnant women receiving IVF combined with latent tuberculosis infection should be large, and its potential risks should be paid enough attention to.

After adjusting for potential confounders, receiving IVF treatment was still associated with adverse perinatal outcomes. This effect was likely mediated by the shorter onset time and more maternal complications (the infant was more likely to be affected by TB before maturation). Spontaneous abortion accounted for 72.2% (13/18) of infant mortality cases in the treatment group. In these cases, vaginal bleeding (6/13, 46.2%) and fallopian tube obstruction (11/13, 84.6%) were common. Abdulhakim et al. reported that approximately 40% of infertility cases with a tuber factor were found to have genital TB (22). Therefore, some cases of spontaneous abortion may have been induced by the reactivation of tubal TB. Lin et al. reported that cured endometrial TB was also associated with a low live birth rate (23). We reviewed the medical records of all IVF-TB cases (including ultrasound, magnetic resonance imaging, computed tomography, and gynecologic examination report findings) to assess the possibility of pre-existed endometrial TB, but the evidence was quite limited.

This study's evidence suggests that women receiving IVF treatment may develop immunosuppression, but this evidence is still insufficient to establish that the adverse outcomes are attributable to IVF-related immunosuppression. In the IVF treatment group, 89.3% of the participants developed miliary TB, and the mean CD4+ T cell level was lower than control group (not statistically significant, likely due to the small sample size). As it is known, miliary TB is characterized by the dissemination of tiny tubercles to one or more organs of the body, often combined with systemic manifestations including high fever, respiratory failure, or meningitis. With a skewed shift toward Th2 in the Th1/Th2 response, TB reportedly tends to develop into a disseminated disease (10, 24, 25). The added progestogen used during IVF may induce increased immunosuppression to the Th1 response and reduce CD4+ T cell proliferation (6), and TB may amplify this side effect into a clinical phenomenon. IVF-TB patients are more likely to develop systemic symptoms and complications. In contrast, pleural effusion, which is considered as localized TB with Th1 predominance (10), was more often observed in control group (36.5 vs. 3.6%). These evidences suggest that IVF may be associated with a damaged host immunity against TB.

Another indirect evidence pointing to the association between IVF treatment and host susceptibility to TB is that most IVF-TB patients, rather the control group patients develop symptoms earlier (14.07 ± 7.38 vs. 22.73 ± 9.31 gestational weeks). In routine practice, progestogen supplementation is often discontinued at the 8–10th gestational week (11, 13), and a large proportion of patients develop symptoms a few weeks later. In contrast, this trend was not observed in the control group. This finding shows a clear intervention point for TB after IVF treatment: in a high-prevalence context, healthcare providers should screen for TB during the 8–22nd gestational week in women who underwent IVF treatment if they present with relevant symptoms. However, over one third of not-IVF participants did not present typical TB symptoms. Performing laboratory tests to rule out TB should be more efficient. Pasipamire et al. reported that routine TB symptom screening is insufficient to rule out TB in pregnant and postpartum women (26). In our study, the routine TB tests, such as sputum acid-fast smear, MTB culture, or IGRAs yielded relatively higher positivity. The IGRAs should be considered as an ideal rule-out tool because almost all participants tested positive on IGRAs at admission.

In this study, participants without a record or confirmation of IVF history were categorized into the control group. If some participants did not report their IVF history, the results might bias towards the null hypothesis.

We adjusted for potential confounders as far as we were aware (2–5, 27). However, due to the limited sample size and retrospective design, it was difficult to adjust for all confounders and verify possible mediating effects (such as the worse perinatal outcome mediated by shorter onset time). Nonetheless, data on IVF-associated TB-in-pregnancy are scarce, and we consider this preliminary study valuable clinical evidence.

This study has some additional limitations. First, the current data do not reveal the number of participants latently infected before IVF treatment because TB screening was not routinely performed; thus, we cannot calculate the risk of new infection and reactivation of TB from receiving IVF treatment. Second, the route of administration and accurate dose of progestogens used in the IVF treatment were not well recorded, thus introducing potential confounders. If some participants in the treatment group did not receive sufficient progestogens, the results would have a negative bias. Third, participants enrolled in this study were all hospitalized patients; therefore, their clinical findings could be different from those of outpatients.

In conclusion, the influence of IVF treatment on host immunity against TB and other pathogens is not sufficiently assessed. Receiving IVF treatment may render women more vulnerable to TB and is associated with worse perinatal outcomes. Investigators should be aware of women preparing to receive IVF treatment in a high-burden TB context, evaluate TB risk, and administer prophylactic therapy for high-risk populations.

## Data availability statement

The raw data supporting the conclusions of this article will be made available by the authors, without undue reservation.

## Ethics statement

The studies involving human participants were reviewed and approved by Ethics Committee of Shanghai Public Health Clinical Center. Written informed consent for participation was not required for this study in accordance with the national legislation and the institutional requirements.

## Author contributions

X-HL and S-HL: conception and design of study. LX and X-HL: acquisition of data. LX, PM, X-HL, Z-DH, and X-YF: analysis and/or interpretation of data.

## Funding

This work was supported by the National Science Foundation of China [Grant Number: 81900005] and Shenzhen Key Medical Discipline Construction Fund [Grant Number: SZGSP010].

## Conflict of interest

The authors declare that the research was conducted in the absence of any commercial or financial relationships that could be construed as a potential conflict of interest.

## Publisher's note

All claims expressed in this article are solely those of the authors and do not necessarily represent those of their affiliated organizations, or those of the publisher, the editors and the reviewers. Any product that may be evaluated in this article, or claim that may be made by its manufacturer, is not guaranteed or endorsed by the publisher.
